# Horses discriminate between facial expressions of conspecifics

**DOI:** 10.1038/srep38322

**Published:** 2016-12-20

**Authors:** J. Wathan, L. Proops, K. Grounds, K. McComb

**Affiliations:** 1 Mammal Vocal Communication and Cognition Research, School of Psychology, University of Sussex, UK; 2 Centre for Comparative and Evolutionary Psychology, Department of Psychology, University of Portsmouth, UK

## Abstract

In humans, facial expressions are rich sources of social information and have an important role in regulating social interactions. However, the extent to which this is true in non-human animals, and particularly in non-primates, remains largely unknown. Therefore we tested whether domestic horses (*Equus caballus*) could discriminate between facial expressions of their conspecifics captured in different contexts, and whether viewing these expressions elicited functionally relevant reactions. Horses were more likely to approach photographic stimuli displaying facial expressions associated with positive attention and relaxation, and to avoid stimuli displaying an expression associated with aggression. Moreover, differing patterns of heart rate changes were observed in response to viewing the positive anticipation and agonistic facial expressions. These results indicate that horses spontaneously discriminate between photographs of unknown conspecifics portraying different facial expressions, showing appropriate behavioural and physiological responses. Thus horses, an animal far-removed from the primate lineage, also have the ability to use facial expressions as a means of gaining social information and potentially regulating social interactions.

Faces are a source of potentially valuable ‘public information’ that can be freely obtained, whether given purposefully or not by the signaller^1^. Faces can have features that are characteristic of age, sex and identity, and this information is processed rapidly and often subconsciously^2^. Facial expressions can also represent another’s internal states (e.g. pain) and consequently accurate perception and recognition of facial expressions has the potential to make behaviour more predictable, facilitating social interactions and bonding^3^. This is particularly important for group living species, where the management of relationships and group cohesion is essential for the maintenance of social networks^4^. However, while many non-human animals demonstrate distinctive facial expressions, some of which clearly resemble those seen and recognised cross culturally in humans^5^,^6^, animal facial expressions have received surprisingly little scientific attention.

Darwin established that a wide variety of animals have the capacity for potentially meaningful facial displays^7^, but few studies have since gone on to directly examine these or uncover their meaning, function, and evolutionary history. Recent research investigating the capacity for facial expression production in primates has revealed that when body size is controlled for (as larger species tend to produce a wider range of discrete facial movements), facial mobility increases in line with group size and complexity^8^,^9^. This corresponds with suggestions that facial expressions are rich sources of social information and that they might have a key role in the management of relationships^10^. However, research investigating non-human facial expressions has been conducted predominantly with primates, and so the true ability of other species remains largely unknown^3^,^11^. New research systematically documenting facial muscles and expressions in non-primates (domestic horses [*Equus caballus*], dogs [*Canis familiaris*] and cats [*Felis catus*]) has demonstrated extensive capacity and a surprising amount of similarity with humans and other primates^12^,^13^,^14^. A range of different facial expressions have also been documented in plains zebra (*Equus quagga*), fur seals (*Arctocephalus forsteri*), and walruses (*Odobenus rosmaru*)^15^,^16^. Clearly, animals other than the primates are able to produce facial expressions, and investigating how these relate to social and ecological variables, could reveal a much broader evolutionary context.

Experimental work examining what information receivers extract from signals, and how this may be functionally relevant, has been neglected in comparison to work on the production of facial expressions^3^,^17^. Captive chimpanzees (*Pan troglodytes*) and some macaques (*Macaca nigra, Macaca mulatta*) can match corresponding emotional facial expressions occurring in different individuals, and chimpanzees can also match a conspecific facial expression to a context of similar valence (e.g. a scream face to scenes of chimps being injected during a veterinary procedure)^18^,^19^,^20^. Touch screen experiments have also demonstrated that crested macaques can predict likely social outcomes from facial expressions^21^, and these are amongst the few studies in non-humans to address questions about the wider perception and significance of facial expressions^3^. Results from non-primates have demonstrated that sheep (*Ovis aries*) discriminate between neutral and stressed faces of conspecifics^22^, and domestic dogs show differing gaze biases when viewing photographs of conspecific facial expressions (suggesting some discrimination between positive and negative valence)^23^. Interestingly, a recent study showed that rats (*Rattus norvegicus*) did not seem to be able to use facial expressions alone to differentiate conspecifics in pain from neutral rats, despite the previous identification of a rat pain face^24^,^25^. This illustrates the importance of investigating not just the production of facial expressions but also how conspecifics respond to them in functionally relevant contexts. Here we use naturalistic approach/avoidance paradigms alongside physiological (heart rate) measurement, to investigate the perception of conspecific facial expressions in a highly social non-primate: the domestic horse.

Horses are gregarious creatures, which form strong and lasting social bonds with conspecifics^26^. When not subject to domestic pressures, horses typically live in large societies comprised of several relatively stable sub-groups that share space and resources in overlapping home ranges^26^. Thus horses regularly come into contact with many other conspecifics, and inter-group dominance indicates that within the larger herd established social relationships exist^26^. Consequently, horses show some degree of fission-fusion dynamics, the same social organisation that is seen in humans, bonobos (*Pan paniscus*), chimpanzees, and macaques (*Macaca spp.*), as well as elephants (*Loxodonta africana, Elephas maximus)*, spotted hyenas (*Crocuta crocuta*), and many cetaceans (*Cetacea spp*.)^27^. In line with this, horses exhibit some of the social skills suggested to be necessary for navigating a fluid and dynamic social environment^28^,^29^: horses can perform individual recognition of social partners^30^,^31^,^32^, they have good long-term memories^33^, they engage in post-conflict consolation^34^, and they intervene in third-party social interactions^35^.

Horses display a wide range of facial expressions and are sensitive to facial cues reflecting the attentional state of conspecifics^12^,^36^,^37^. We conducted two experiments to investigate how horses responded to stimuli of conspecific faces captured in different contexts. First, we presented horses with the opportunity to investigate two photographs of unfamiliar conspecific faces displaying different expressions in a free-ranging approach/avoidance paradigm. The expressions depicted were captured opportunistically when (i) the subjects were anticipating food (positive attention), (ii) in a relaxed context, and (iii) in an agonistic context (Fig. 1). We then further explored the behavioural and physiological responses to two of the expressions in a second experiment, where horses viewed the positive attention and agonistic stimuli in single presentations.

## Study 1

### Methods

Forty-eight horses from stables in Merseyside, Sussex, and Surrey, UK participated in this experiment: 29 gelded males and 19 mares (3–32 years, *M* = 15.04, *SD* = 6.03). Each horse participated in one trial, in which they were lead into an indoor arena where they were given the opportunity to interact with two photographs of an unfamiliar horse displaying different facial expressions (see below for test procedure). The two photographic stimuli presented one pairwise comparison of the same horse captured in two different contexts (either positive attention/agonistic, relaxed/agonistic, or positive attention/relaxed). The facial expressions produced by our models corresponded with other records of horse facial expressions given in these contexts and we use the existing nomenclature for the expressions as applied in established ethograms^26^,^38^ (see Table 1). We also describe the discrete facial movements captured in the stimuli using the Equine Facial Action Coding System (EquiFACS)^12^ to facilitate objective and direct comparison with other species and studies. Two certified EquiFACS coders characterised the expressions, one of whom was blind to the context of the stimuli, purpose of the stimuli, and the research being conducted. There was 96% agreement on the codes. Each expression was presented on the left and the right of the experimental set-up an equal number of times across the trials (see Additional Methods for full details of stimuli preparation).

The two photographic stimuli were attached to the wall 1.5 m apart at the inner edges and 0.4 m from the floor at the bottom edge. Jump poles were placed 1 m away from the outer edge of the photographs, perpendicular to the wall. These were raised on a plastic block at one end and fanned out by 0.5 m at the other end to create a definable testing area. Participating horses were brought into the arena by an experimenter, and led in a figure of eight before turning and being released 3 m away from the photographs. After releasing the horse, the experimenter leading the horse walked away and stood directly behind the horse facing away from the experimental set up so she could not see the horse’s behaviour or provide any cues. See Fig. 2 for a diagrammatic representation of the experimental set up, and Supplementary Video S1 for a full example of a trial.

Upon release horses were allowed to enter the testing area and interact freely with the photographs. The end of the trial was defined as when the horse chose to leave the testing area, up to a point 120 s from release; if the horses were still in the testing area at this time they were caught and the trial was ended unless they were still actively investigating the photograph, in which case they were allowed to finish their investigations before the end of the trial. If upon release horses immediately left the test area they were caught, walked in another figure of eight, and released again. Horses that did not approach the set up after 3 releases were counted as non-responders. Trials were video recorded on two video cameras placed at different viewpoints (see Fig. 2, cameras were either a Canon XM2 and Sony DCR-SR58E handycam or Panasonic HC-V720 and X920 handycams). For each trial we coded whether the horse approached either of the stimuli, and if so which stimuli they approached first. We also coded time spent looking at the photographs, time stood in proximity to the photographs, and time spent touching the photographs (for more detailed procedures see Additional Methods below). All data supporting this study are provided in the Supplementary Data File.

### Results

Horses approached the positive attention stimuli significantly more often than the agonistic stimuli (*n *= 11, *K *= 11, *P *< 0.001), and the relaxed stimuli significantly more than the agonistic (*n *= 12, *K *= 10, *P *= 0.04). However, horses did not discriminate between the positive attention and relaxed stimuli (*n *= 13, *K *= 6, *P *> 0.99) (Fig. 3). Horses also preferred to look at the positive attention stimuli over the agonistic (*Mdn *= 8.91 s vs. *Mdn *= 2.98 s, *Z *= −2.74, *P *= 0.006, *r *= 0.48) and the relaxed stimuli compared to the agonistic (*Mdn *= 8.41 s vs. *Mdn *= 2.40 s, *Z *= −2.53, *P *= 0.01, *r *= 0.45), although they showed no preference for looking at the positive attention versus the relaxed stimuli (*Mdn *= 4.53 s vs. *Mdn *= 5.18 s, *Z *= −0.11, *P *= 0.91). Furthermore, horses chose to spend more time in front of the positive attention stimuli than the agonistic (*Mdn *= 24.48 s vs. *Mdn *= 1.29 s, *Z *= −2.72, *P *= 0.007, *r *= 0.48), and the relaxed stimuli than the agonistic (*Mdn *= 15.42 s v.s *Mdn *=  0 s, *Z *= −2.33, *P *= 0.02, *r *= 0.41), however again showed no difference in the time they stood in front of the positive attention and relaxed stimuli (*Mdn *= 11.87 s vs. *Mdn *= 11.84 s, *Z *= −0.66, *P *= 0.51). In addition, when horses were stood in front of the positive attention stimuli they were significantly more likely to spend time in close proximity to the photograph (<1.5 m) than further away (>1.5 m) (n* *= 24, *Mdn *= 24.17 s vs. *Mdn *= 3.47 s, *Z *= −3.26, *P *= 0.001, *r *= 0.46). When horses were stood in front of the relaxed stimuli they also chose to spend time in close proximity to the image rather than further away (n* *= 25, *Mdn *= 13.44 s vs. *Mdn *= 2.43 s, *Z *= −2.61, *P *= 0.009, *r *= 0.37). However, this was not seen for the agonistic stimuli, where horses showed no preference for the half of the testing area containing the stimuli compared to further away (n* *= 14, *Mdn *= 6.50 s vs. *Mdn *= 2.58 s, *Z *= −0.91, *P *= 0.36).

To investigate whether other factors were influencing the behaviour (looking time and proximity to the stimuli) of horses in our trials, we ran a series of General Linear Mixed Models in which we entered age and sex of the participating horse, the model horse that they were exposed to, the pairwise comparison they were exposed to, which stimulus they were engaging with, and side of stimulus presentation as fixed factors, and the identity of the horse as a random factor (See Additional Methods and Supplementary information for full details of model selection). The expression displayed in the photograph was a significant parameter and was featured in all of the top models (Supplementary Tables 2 and 3). This confirmed the results of the initial analysis (see above), demonstrating that horses showed no preference in proximity or looking time between the positive attention and relaxed expressions, but chose to stand in front of and look at the agonistic expression significantly less (Supplementary Table 2). Younger horses spent more time overall looking at the photographs, but there was no interaction between age and expression, suggesting that younger horses were generally more vigilant. Age did not significantly influence the horses’ proximity to the expressions, and the 95% confidence intervals overlapped zero for all other potential predictors, indicating that they had no effect on looking behaviour or proximity to the stimuli (Supplementary Table 2).

## Study 2

In order to measure more detailed behavioural and physiological reactions to the stimuli, in study 2 the stimuli were presented to horses in single presentations in a more controlled environment. In study 1, the horses had chosen to approach the images taken in the positive attention and relaxed contexts, and generally avoided the agonistic image. As responses to the relaxed stimuli in study 1 were not significantly different from the positive attention stimuli and to reduce potential habituation effects, in study 2 each participating horse received only two oppositely valenced stimuli: one positive attention and one agonistic image of the same horse.

### Method

Thirty-three horses were recruited from stables in Sussex, UK: 14 mares, 19 gelded males (5–27 years, *M *= 16.81, *SD *= 4.34). Each horse participated in two trials in which they were presented with a photographic stimulus (positive attention or agonistic counterbalanced across trials) for a total of 30 seconds. An experimenter initially held the stimulus up at a point 1 m from the horse’s nose for 10 seconds, then slowly moved it forward 0.1 m and held it there for 10 seconds, before moving it back to the starting position for a final 10 seconds. To ensure accurate presentation, chalk lines on the stable floor marked the positions for the stimulus presentation and the starting position of the horse. A second experimenter held the horse on a 1.5 m long rope and allowed the horse to move freely within the range of the rope during the trial. The experimenter holding the horse stood approximately at the horse’s shoulder, facing the rear of the animal so that she could not see the stimulus and incidentally cue the horse. The stimuli were presented so that the top of the picture was level with the horse’s withers, and the experimenter conducting the presentation kept their head hidden behind the stimulus. A third person arranged the presentation to ensure both experimenters were blind to the stimulus being presented, and therefore any potential ‘Clever Hans effect’ could be discounted. See Fig. 4 for a diagrammatic representation of the experimental set up, and Supplementary Video S2 for an example trial.

Time between presentations was 61–167 days (*M *= 81.18, *SD *= 23.17). Heart rate was monitored for 5 minutes prior to the presentation to gain an average resting baseline for the horse, for the duration of the presentation, and then for 5 minutes immediately post-test to monitor recovery rate. Heart rate was recorded using a Polar Equine RS800CX heart rate monitor (see Supplementary Material for details of heart rate data processing). An experimenter remained with the horse during the pre and post-test recording time, but did not interact with the horse except to prevent excessive movement. For the heart rate analysis, data for one horse were lost due to equipment failure and five horses were removed due to a high proportion of corrections in one of the heart rate recordings (>5%)^40^, leaving 27 horses in the final analysis (12 mares, 16 gelded males; 5–27 years, *M *= 16.82, *SD *= 4.64). Three further horses were removed from the recovery analysis because their heart rate did not actually increase during one of the trials and thus recovery measures were not appropriate.

Trials were video recorded from two viewpoints on Panasonic HC-V720 and X920 handycams with a wide view lens attachment. For each presentation, we coded looking time at the stimulus. This was split into three measures: direct looking, left gaze bias (i.e. head turned to the right to give the left eye precedence), and right gaze bias (head turned left to give the right eye precedence). We also measured all approach and avoidance behaviours, whether horses touched the stimulus, and their ear position throughout the trial. All data supporting this study are provided in the Supplementary Data File.

### Results

#### Behavioural responses

In line with study 1, horses spent significantly more time actively avoiding the agonistic stimuli than they did the positive attention, with very few avoidance behaviours to the positive attention stimuli (*Mdn *= 2.73 vs. *Mdn *=  0, *Z *= −2.11, *P *= 0.03, *r *= 0.26). More horses (*n *= 12) engaged in approach behaviours to the positive attention stimuli compared to the agonistic stimuli (*n *= 5), but time spent approaching the stimuli overall was low and statistical comparison was not valid on this small dataset.

There was no difference in direct binocular looking time to the positive attention and agonistic stimuli (*Mdn* = 23.02 vs. *Mdn *= 22.81, *Z *= −0.36, *P *= 0.72). When each eye is considered in isolation, there was also no difference in time spent viewing the positive attention and agonistic stimuli (Left gaze: *Mdn *= 2.57 vs. *Mdn *= 2.82, *Z *= −0.94, *P *= 0.36; Right gaze: *Mdn *= 0 vs. *Mdn *= 0.32, *Z *= −0.11, *P *= 0.92). However, there was an overall preference for viewing both stimuli with the left eye versus the right eye (*Mdn *= 2.64 vs. *Mdn *= 0.16, *F*_1, 128_* *= 9.44, *P *= 0.003). Although the interaction between emotional stimulus and gaze bias was not in itself significant (*F*_1, 128_* *= 0.65, *P *= 0.42), pairwise contrasts with adjusted significance revealed that when viewing the agonistic stimuli, horses displayed a preference for using their left eye compared to their right eye (*Mdn *= 2.82 vs. *Mdn *= 0.32, *F*_1, 128_* *= 5.88, *P *= 0.017). This was not significant when viewing the positive attention stimuli, but the p value was approaching significance (*Mdn *= 2.57 vs. *Mdn *= 0, *F*_1, 128_* *=  3.57, *P *= 0.061). Horses spent the same amount of time with their ears forward when viewing the positive attention stimuli and the agonistic stimuli (*Mdn *= 21.6 vs. *Mdn *= 20.52, *Z *= −0.71, *P *= 0.48), but more time with both ears angled backwards when viewing the positive attention stimuli compared to the agonistic stimuli (*Mdn *= 1.32 vs. *Mdn *= 0.23, *Z *= −2.4, *P *= 0.02, *r *= 0.30. Horses spent longer with their ears in an asymmetrical position (one forward one back) when viewing the agonistic stimuli than when viewing the positive attention stimuli (*Mdn *= 5.4, vs. *Mdn *= 3.8, *Z *= −2.4, *P *= 0.02, *r *= 0.30), however there was no preference for a specific asymmetrical ear combination in response to either of the stimuli (*P* > 0.05 for all comparisons). Only 4 horses touched the stimuli so no analysis was performed.

To investigate whether other factors were influencing the behaviour (direct looking and avoidance time) of horses in our trials we ran a series of General Linear Mixed Models, in which we entered age and sex of the participating horse, which model horse they were exposed to, which stimulus they were presented with, and the trial number (first or second) as fixed factors, and the identity of the horse as a random factor (See Additional Methods and Supplemental for full details of model selection). The GLMM analysis confirmed our initial results (see above), showing that the facial expression presented significantly influenced the time horses spent engaging in avoidance behaviours but did not influence the time that horses spent (directly) looking at the expressions (see Supplementary Tables 4 and 5). Horses also spent significantly longer looking at model horse 1 than model horse 2 (horse 1 *M *= 24.34, *SEM *= 0.85; horse 2 *M *= 19.34 *SEM *= 1.42), although there was no significant difference in time spent avoiding the model horses (horse 1 *M *= 1.60, *SEM *= 0.40; horse 2 *M *= 4.40, *SEM *= 1.08). Male horses looked at the stimuli for longer than females (males *M *= 23.83, *SEM *= 1.03; females *M *= 19.68, *SEM *= 1.33) but females were more reactive and spent more time avoiding the stimuli than males (males *M *= 1.73, *SEM *= 0.51; females *M *= 4.42, *SEM *= 1.08). Finally, horses spent significantly more time avoiding the stimuli in the first presentation than the second presentation (first *M *= 4.14, *SEM *= 0.97; second *M *= 1.6, *SEM *= 0.50); however they spent more time looking at the stimuli in the second presentation than the first (first *M *= 20.39, *SEM *= 1.26; second *M *= 23.78, *SEM *= 1.08).

#### Heart rate

In response to the stimuli captured in the context of positive attention, the modal heart rate decreased slightly between the baseline period and the test period, whereas there was a slight increase when viewing the agonistic stimuli, with the difference bordering on significance (*Mdn *= −1 vs. *Mdn *= 1, *Z *= −1.94, *P *= 0.052, *r *= 0.26). In line with this, horses took less time to return to their baseline mode (measured from the end of the test) after viewing the positive attention expression than after viewing the agonistic expression (*Mdn *= 5.2 vs. *Mdn *= 30.1, *Z *= −2.02, *P *= 0.044, *r *= 0.29). The raw data for experiments 1 and 2 are available as Supplementary Material (Data File S1).

### Discussion

Our results show that horses can spontaneously distinguish between facial expressions of conspecifics captured in different contexts, and that viewing these expressions stimulates functionally relevant behavioural and physiological responses. In our experiments the photographs of facial expressions produced in positive attention and relaxed contexts were clearly discriminated from facial expressions produced in agonistic contexts, with the positive attention/relaxed expressions eliciting more approach behaviours and the agonistic expressions more avoidance behaviours. Such reactions are likely to be highly adaptive if horses were to encounter a conspecific displaying these facial expressions, potentially reducing the probability of conflict and increasing the opportunity for affiliative interactions.

Our physiological data corresponds with this, demonstrating that when horses viewed the agonistic expressions, modal heart rate increased slightly whereas it decreased slightly for the positive stimuli. Heart rate also took longer to recover when viewing agonistic expressions. Increased heart rate can be a physiological preparation for impending escape or defensive behaviour^41^,^42^, which again may be adaptive if horses encounter conspecifics displaying agonistic intent. In contrast, lower heart rate tends to reflect an increased state of relaxation. Heart rate is also associated with arousal^43^ and the potential influence of this must be considered when interpreting the results; however, as positive anticipation and agonistic behaviour are both high arousal states they might be expected to have similar effects in this regard. More generally, it is important to consider the heart-rate results in the wider context of the associated behavioural data. In this study the agonistic stimuli produced both a slight raise in heart rate and an increase in avoidance behaviour, whilst the positive attention stimuli produced a slight drop in heart rate and increased approach behaviour. Therefore, these results provide an interesting first insight into this, but are very preliminary – possibly due to the short time window they were collected in. More detailed studies, that collect enough data to include heart rate variability, will be important in future to help us understand how animals perceive these expressions and the physiological mechanisms that underpin their behaviour.

Ear responses have been linked to emotional states in animals with mobile ears, and the patterns of ear movements demonstrated by horses in our experiments corresponds with previous work in this area. In our study, horses spent significantly more time with their ears in an asymmetrical position when viewing the agonistic photographs than when viewing the photographs of positive attention – a pattern of behaviour that is associated with negative emotional experiences in horses^44^, sheep^45^,^46^, rats^24^, and mice^47^. Perhaps surprisingly, when viewing the positive facial expression there was a tendency to hold the ears back, which is generally associated with negative affect (e.g. refs 26 and 44). However, in sheep holding both ears backwards and holding the ears asymmetrically are associated with subtly different experiences^45^. As it has been documented that horses have a range of different ear movements^12^ more detailed studies of the extent of ear movements are needed to fully elucidate how ear position and movement may be associated with affective state.

It is possible that the horses were reacting to one obvious cue in the stimuli, such as ear position, rather than a suite of facial cues making up an overall ‘expression’. As there is so little previous research investigating functionally relevant responses to facial expressions it was our intention to provide the most complete naturalistic stimuli possible, and so our images contain a range of appropriate cues. In a previous paper we found that preventing access to information from the eye region and the ear region both had detrimental effects on the ability of horses to gain information from conspecific signals^36^. Therefore, it seems likely that both of these facial areas (along with possibly others, such as the mouth) are informative. A detailed investigation of the role of different cues would be an interesting avenue for future research. It should also be considered that the stimuli presented in our study depicted static facial expressions, without the dynamic cues that would be present in real interactions. Whilst our method offers a level of control, novel experimental approaches, potentially incorporating video images, could be used to explore dynamic signal elements in the future.

With advances in digital photography, the use of two-dimensional (2D) images to present visual animal models has become a common and successful method for studying face processing across species (e.g. Refs 18, 22 and 48). However, it is still critical to ensure that responses to photographic images of conspecifics adequately represent those seen during live interactions with real conspecifics. When responding to our photographic stimuli, horses gave ecologically valid responses that suggested the photographs were being interpreted as horse stimuli. For example, we observed behavioural reactions corresponding to the greeting behaviour typically observed when horses encounter conspecifics (e.g. nose to nose contact)^26^,^39^. Also, the responses seen to our stimuli were markedly different from typical responses of horses to novel objects^49^. This corresponds with a previous study in which we found significant differences in the behavioural responses of horses to photographic stimuli of conspecific faces and phase scrambled versions of the same images, presented under identical protocols^36^. Furthermore, previous evidence has demonstrated that horses are able to associate 2D photographs of inanimate objects with their 3D counterparts^33^, and horses that learn to discriminate between rewarded and unrewarded human faces in photographs spontaneously transfer this knowledge to the real people when they appear in a novel setting^50^.

Horses have almost complete decussation of the optic nerves (80–90%) suggesting that behavioural asymmetries reflect asymmetries in hemispheric activation^51^. Across mammals the right hemisphere is generally specialised for novel and emotional stimuli, and in particular the assessment of threatening situations^52^,^53^,^54^. In humans, the right hemisphere is also thought to be predominant in facial processing and identity recognition^52^. When examining reactions to the facial expressions as a whole, the horses in our study showed an overall gaze bias for using the left versus the right eye, prioritising the right cerebral hemisphere. However, when examining reactions to each expression in isolation, it is notable that only the agonistic expression generated a significant preference for viewing the stimulus with the left eye. The overall right hemispheric dominance displayed in our studies could reflect the activation of face processing centres, which may potentially have been further enhanced when subjects were presented with the threatening negative facial image. However, the lack of a significant interaction between stimulus emotion and gaze bias to positive attention and agonistic expressions means that this result should be interpreted cautiously.

In experiment 1 of this study, younger horses spent longer looking at the photographs compared to older horses. This is consistent with previous reports that younger horses spent more time looking at a novel object than older individuals and may reflect the increased time required by less experienced individuals to evaluate the situation^55^. Age and experience also improves the recognition and appraisal of social cues in humans^56^ and African elephants^57^,^58^. Sex also influenced the behaviour of horses in experiment 2 of our study; males spent longer looking at the stimuli, regardless of emotion portrayed, whereas females were more reactive (spent more time avoiding the stimuli). This may potentially reflect the different roles the sexes play in free-ranging horse societies, where males take on a protective role for the group^26^.

It is notable that the reactions displayed by horses in our experiments were common to both the model horses used as stimuli, and our ongoing research is successfully expanding the size of the stimulus set to increase the range of individual variation encompassed (Proops/Grounds/McComb, unpublished data). The use of photographic models, therefore, offers a way in which visual signals can be systematically presented to examine what information receivers gain and how this might influence behaviour – a neglected aspect in the study of facial expression^3^. The responses of horses to the photographic stimuli in our experiments support the idea that facial expressions have a socially facilitative purpose in non-human animals, providing important information about conspecifics, and potentially making their behaviour more predictable.

Recent evidence has demonstrated an extensive capacity for production of facial expressions in the horse, with a surprising number of similarities in structure and form to some primates^12^. In our experiments, although horses distinguished the expressions captured in positive attention and relaxed contexts from those produced in agonistic contexts, they did not show a clear behavioural discrimination between positive attention and relaxed. This has also been seen in chimpanzees, who did not discriminate a positive (relaxed lip) face from a neutral face, even though they could distinguish these faces from other expressions^18^. Potentially, in our experimental situation – determining whether to approach or avoid an unfamiliar conspecific – there may be no functional value in differentiating between expressions associated with positive anticipation and a relaxed state. However, this result might also lead us to question whether such expressions have differing representations in non-human animals – indeed, even in humans relaxation and contentment elicit the same beneficial effects on the cardiovascular system as mild joy^59^,^60^. This highlights the need to better understand and characterise animal emotional experiences (and positive experiences in particular), and address the growing interest in what emotions non-human animals have and how these are expressed^61^,^62^.

To summarise, our results show that horses gain important information from the facial expressions of others, which influences their physiology and behaviour in functionally relevant ways. Our findings support recent calls for scientists to extend their studies of the production and perception of facial expressions across a wider range of species. This would allow us to consider more fully the extent to which some facial expressions may result from shared ancestral characteristics or may offer some advantage in response to common selective pressures. To progress, we also need to devise innovative paradigms that elucidate the cognitive, neurological, and physiological mechanisms underpinning responses to facial expressions and examine their corresponding fitness benefits. Moreover, future research more directly exploring the link between facial expressions and animal emotional experiences could be very valuable. A better understanding of what facial expressions mean, particularly those associated with emotion, could also have significant impacts on the management of captive, domestic, and even wild animals.

## Additional methods

### Experiment 1 participants

An additional ten horses were tested but excluded because of a side bias at one centre (9/10 horses went left, towards the entrance to the stable block; two tailed binomial probability, *P *= 0.02).

#### Stimuli

**Figure 1:**
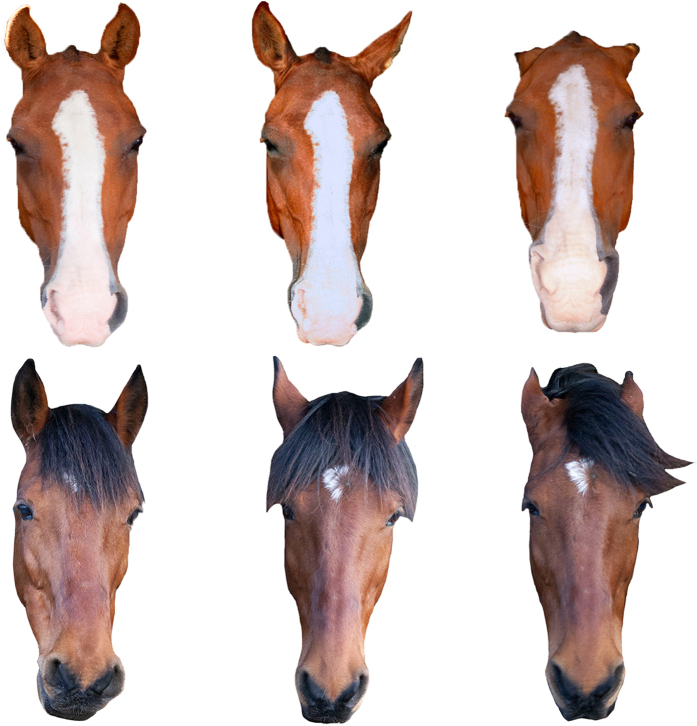
Photographic stimuli used in both experiments. Facial expressions shown from left to right are positive attention, relaxed and agonistic. Horse 1 is displayed at the top of the image and horse 2 at the bottom of the image. We credit and thank Katie Slocombe for the photographs of horse 1 and Amy Lyons for those of horse 2.

**Figure 2:**
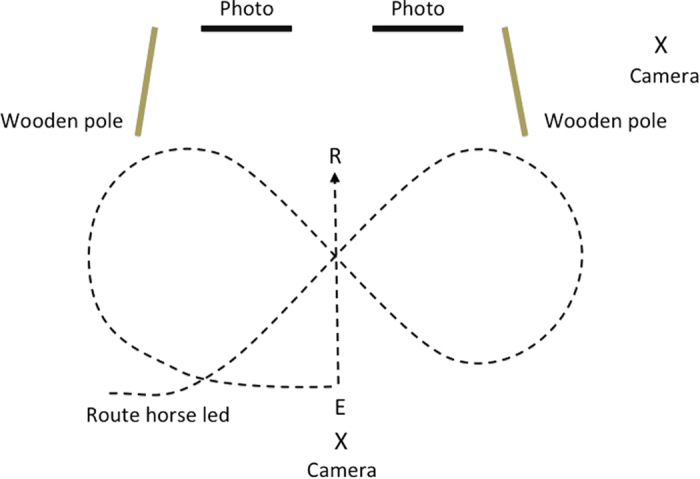
Diagrammatic representation of the experimental set up of experiment 1. X refers to the position of the cameras, R to the release point, and E to the position of the experimenter after release.

**Figure 3:**
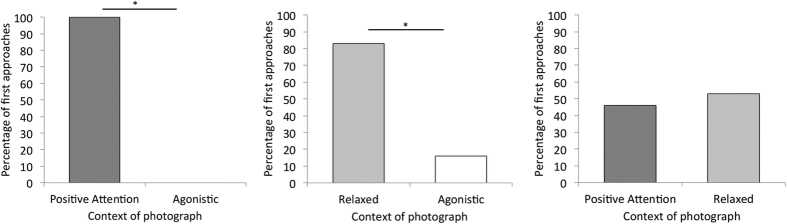
Percentage of horses choosing to make their first approach to each photographic stimulus in a series of paired choice tests. *Represents a significant difference.

**Figure 4:**
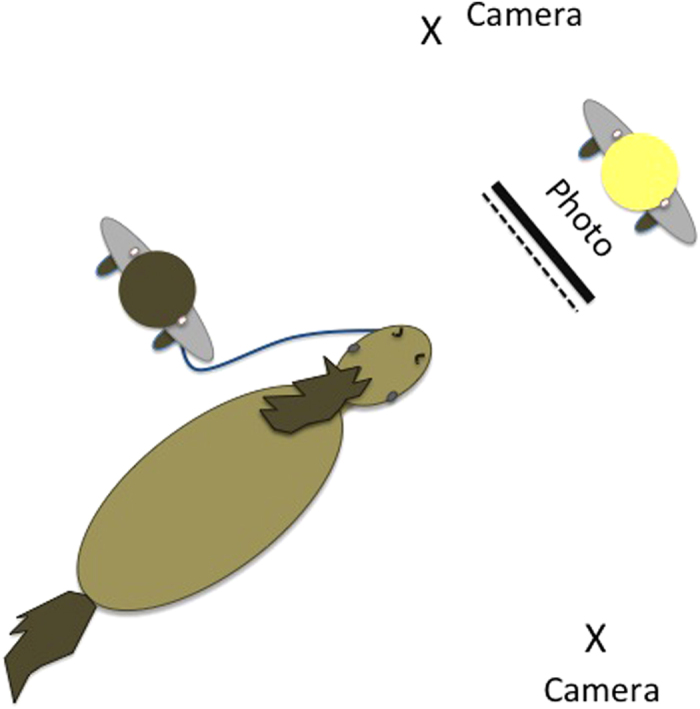
Diagrammatic representation of the experimental set up of experiment 2. The dotted line represents the markers on the floor used by the experimenter to ensure correct positioning of the stimulus being presented.

High quality photographs were taken of two horses (one adult female, age 10 years; one adult gelded male, age 9 years) in different contexts: a ‘positive’ situation, where horses were receiving and anticipating the delivery of a food reward and were attentive to the handler preparing the food (positive anticipation), when the horses were relaxed and sleepy, and a negative, agonistic situation, where one horse was having her underside touched and the other had been left in the stable while his stable-mates were taken out to the field (aversive situations for these horses, Fig. 1). Photographs were taken with a Canon 350D DSLR camera. Three photographs of each horse (one from each context) were selected to create the final stimuli set. The images were then extracted, placed onto a white background, and auto adjusted for levels and brightness in Adobe Photoshop. The stimuli were enlarged to A1 (841 × 594 mm), printed and laminated.

#### Ethical statement

This work complies with the Association for the Study of Animal Behaviour guidelines for the use of animals in research and received approval from the Ethical Review Committee at the University of Sussex. Owners/carers of the horses gave consent prior to participation. Horses were not food deprived and remained in their familiar environment during participation in the study. In experiment 1, 22 horses were brought into the arena but chose not to participate (i.e. did not look at or approach the experimental set up and walked away immediately upon release). Whilst it is not common practice to report the number of participants that decline to participate in an experiment, we report it here for potential replication purposes.

#### Behavioural analysis

Videos from two viewpoints were synchronized and analysed for behavioural responses using Sportscode Gamebreaker Plus (www.sportstech.com). See Table 2 for definitions of the behaviours used for coding. In experiment 1, ten videos (21%) were analyzed by a second coder, revealing good to excellent levels of agreement: did the horse approach a photo (Cronbach’s alpha* *= 1); which photo was approached first (Cronbach’s alpha* *= 1); looking time at the photographs (two measures per trial; Spearman’s rank correlation: *n *= 20, *r* = 0.90, *P *< 0.001); time touching each photograph (two measures per trial; Spearman’s rank correlation: *n* = 20, *r* = 0.94, *P *< 0.001); proximity in each quarter of the test area (four measures per trial; Spearman’s rank correlation: *n* = 40: *r *= 0.85, *P *< 0.001). In experiment 2 the video coding was split equally between KG and JW: KG was aware of the photograph in the presentation, while JW was blind to the stimuli (achieved by using tools in the video software to occlude the stimuli on the screen). Twelve videos (22%) were double coded, revealing good to excellent levels of agreement measured by Spearman’s rank correlation: looking direction (forward, left bias, right bias – three measures per trial), *n* = 36, *r *= 0.99, *P *< 0.001; approach behaviours, *n *= 12, *r *= 0.99, *P *< 0.001; avoidance behaviours, *n* = 12, *r *= 0.93, *P *< 0.001; ear position (both ears forward, both ears backward, left ear forward/right ear backward. Right ear forward/left ear backward – four measures per trial), *n *= 48, *r *= 0.99, *P *< 0.001.

#### Statistical analysis

In experiment 1, which photograph horses approached first (if any) was assessed using two-tailed binomial tests. In 12 trials the horse entered the testing area but did not approach a photograph (positive/negative, *n *= 5; neutral/negative, *n *= 4; positive/neutral, *n *= 3). Data for looking time and proximity were excluded for one horse in the positive attention-relaxed comparison because his trial was cut short (after approaching a photograph) due to equipment failure. The looking behaviour and proximity measures were positively skewed and so were analyzed using Wilcoxon Signed-Rank tests. Although we measured time touching the photographs, too few horses touched the negative photographs (*n *= 5 across all trials) to make statistical comparisons.

In experiment 2 Wilcoxon Signed-Rank tests were also used to analyze whether the emotion displayed in the photographic presentations influenced the behavioural measures and two measures of heart rate: the difference in the average heart rate between the baseline and test period, and the time from the end of the test for the heart rate to return to baseline mode (see Supplementary Material). We chose to examine the mode rather than the median or the mean, as the mode is influenced less by extreme values commonly seen in heart rate recordings. In order to compare the amount of time spent looking left to the amount of time spent looking right, both as a main effect across stimuli and also as an interaction with stimulus type, we also ran a GLMM. Pairwise contrasts were performed to assess the extent of lateralisation for each stimulus type independently. All p-values presented are two-tailed.

We also employed GLMMs to investigate potential effects of subject age and sex, model horse, and presentation order on behavioural measures (although the data were non-normal, visual examination of the residual plots showed satisfactory model fit). Model averaging was conducted to extract parameter β estimates and their 95% confidence intervals. The significance of predictor variables was assessed by whether the 95% confidence intervals overlapped zero. See Supplementary Material for full details of model selection.

## Additional Information

**How to cite this article**: Wathan, J. *et al*. Horses discriminate between facial expressions of conspecifics. *Sci. Rep.* **6**, 38322; doi: 10.1038/srep38322 (2016).

**Publisher's note:** Springer Nature remains neutral with regard to jurisdictional claims in published maps and institutional affiliations.

## Supplementary Material

Supplementary Video S1

Supplementary Video S2

Supplementary Information

## Figures and Tables

**Table 1:** Facial actions present in the photographic stimuli used in our study (as described using EquiFACS) alongside behavioural descriptors for these actions documented in previous research*.

Context	Behavioural Descriptors	Model horse	EquiFACS codes
Positive (forward) Attention	Ears are up and rotated forward. Eyes are directed forward. The nostrils are moderately dilated. The mouth is usually closed^26^,^38^.	1	Ears forward (EAD101)
		2	Ears forward (EAD101)
			Nostril dilation (AD38)
Relaxed	Body and facial muscles are relaxed. Some eye closure. Ears may continue to move or, if very relaxed, will rest in a lateral position. Lower lip may droop^26^.	1	Right ear forward (EAD101, R)
		2	No movements present (0)
Agonistic	Muscles are tense. Ears are rotated backwards and flattened to the skull. Nostrils are usually dilated and drawn back/upwards, causing wrinkles along the upper posterior edge. Lips are often pursed. In more extreme cases, mouth may be open and incisors exposed in a bite threat^26^,^38^.	1	Ear flattener (EAD103)
			Ear rotator (EAD104)
			Nostril lift (AUH13)
			Lip pucker (AU18)
		2	Ear flattener (EAD103)
			Ear rotator (EAD104)
			Nostril lift (AUH13)
			Lip pucker (AU18)

*See also^39^ for photographs and line drawing of these expressions.

**Table 2:** Definitions of behaviours used for coding.

Behaviour	Definition	
**Experiment 1**		
Looking at photograph	Attentive with head orientated directly towards the photograph.	
Approach	Horse walks up to the photograph, bringing their nose to within 30 cm. If horses approached both photographs we recorded which they approached first.	
Touch the photograph	Horse touches the photograph with their nose.	
Time spent in proximity to a photograph	Testing area divided into quarters. Horses’ position measured from the time the first foreleg is placed into the quarter until the first foreleg leaves the quarter.	
**Experiment 2**		
Looking at photograph	Attentive with head orientated directly towards the photograph.	
Gaze bias left/right	Oriented to the stimuli (judged by at least one ear still oriented towards the photograph) but with the head turned to one side. Gaze bias left refers to a preference for viewing with the left eye (i.e. head turn right) and gaze bias right a preference for the right eye (head turn left).	
Avoidance	Horse moves away from the photograph with visible alarm, e.g. nostril flaring, wide eyes, tense muscles.	
Approach	Horse reaches their nose or moves their body forward towards the photograph.	
Touch the photograph	Horse touches the photograph with their nose.	
Both ears forward	The openings of both ears are facing forward of the midline.	
Both ears back	The openings of both ears are facing posteriorly of the midline.	
Asymmetrical ears	Left ear forward/right ear back	The opening of the left ear is facing forward of the midline while the right ear opening is directed posteriorly.
	Right ear forward/left ear back	The opening of the right ear is facing forward of the midline while the left ear opening is directed posteriorly.
